# Aesthetic Experience of Representational Art: Liking Is Affected by Audio-Information Naming and Explaining Inaccuracies of Historical Paintings

**DOI:** 10.3389/fpsyg.2021.613391

**Published:** 2021-07-19

**Authors:** Manuel Knoos, Manuela Glaser, Stephan Schwan

**Affiliations:** Leibniz-Institut für Wissensmedien (IWM), Tübingen, Germany

**Keywords:** liking, aesthetic experience, historical inaccuracies, representational art, audio-text

## Abstract

Paintings in museums are often accompanied by additional information, such as titles or audio-texts. Previous research has reported mostly positive effects of additional information on the liking and subjective understanding of a painting. However, some studies have also reported negative effects when additional information introduces inconsistencies between the painting’s content and the represented reality. Therefore, the present study examined the negative effects of naming a painting’s historical inaccuracies, which are inconsistencies between the content of the painting and the real historic event, and whether these negative effects can be compensated by an explanation for the inaccuracies. The results revealed that liking was lower with inaccuracies named and that this effect was compensated by an explanation for the inaccuracies. No significant effects were observed for subjective understanding and aesthetic emotions. The results corroborate parts of the Vienna integrated model of art perception and have practical implications for the design of audio-texts in museums.

## Introduction

Artworks in museums are often presented together with additional information, such as titles, text labels, or oral explanations in the form of personal or audio guides. In the past years a number of studies focused on the effects of titles on the aesthetic experience of paintings but did not examine the effects of longer additional information such as accompanying audio-texts. However, since art- and art-history museums do not change the titles but frequently provide their visitors with audio guides that include longer explanations of the paintings, examining the viewer’s aesthetic experience of paintings in combination with longer accompanying audio-texts is both of theoretical and practical relevance. Such audio-texts are intended to educate the viewers, to help them to understand the artworks and thereby enhance the visitors’ aesthetic experience in the gallery. They thus differ from other additional information, such as information about the prices of paintings or opinions of other people that could also influence the aesthetic experience, but in a different way, namely via priming the viewers expectations (Lauring et al., 2016). Research corroborates positive effects of titles and short text labels on the viewer’s subjective understanding of paintings (Russell, 2003; Leder et al., 2006; Swami, 2013; Bubić et al., 2017) and their aesthetic appreciation (Millis, 2001; Russell, 2003; Belke et al., 2010; Swami, 2013; Gerger and Leder, 2015; Bubić et al., 2017). However, a recent review (Chmiel and Schubert, 2019) points out a substantial number of studies that did not observe effects of additional information on subjective understanding and aesthetic appreciation in the form of liking. Other research even shows negative effects of mismatching titles on liking (Belke et al., 2010; Gerger and Leder, 2015). For this reason, conditions need to be specified when and how additional information related to an artworks’ meaning influences subjective understanding and liking of artwork as two main aspects of the aesthetic experience (Leder et al., 2004).

First, the effects of titles on subjective understanding and liking mostly apply for abstract rather than for representational art (Chmiel and Schubert, 2019). This has been corroborated by several studies showing effects for highly abstract but not for representational art (Leder et al., 2006; Moore and West, 2012; Swami, 2013). This could indicate, that the iconicity of representational art could provide the viewers with a feeling of an easy and high understanding, whereas abstract art needs clarification of what the painting represents. The absence of effects of additional information on the subjective understanding and liking of representational art might thus be due to the viewers feeling of an already highly subjective understanding and liking even if no additional information is provided.

Second, effects of additional information on the subjective understanding and liking of artwork may depend on the type of the additional information. Comparing descriptive and elaborative titles to a control group without titles, an experiment (Leder et al., 2006) revealed that both titles improved the subjective understanding of paintings compared to the control group. Elaborative titles had the highest effect on subjective understanding but neither of the titles increased liking. Comparing title, broad genre information, and content specific information to a control group without additional information, an experiment (Experiment 1 of Swami, 2013) found that all three types of information improved the subjective understanding of abstract paintings compared to the control condition. In this experiment, content specific information had the highest effect and was the only type of information that improved liking. In addition, the type of additional information can influence whether the additional information affects the liking of a painting positively or negatively. Studies show that paintings are liked more when the provided title semantically matches the content of the painting than if the title does not semantically match the content and this mismatch remains unexplained (Belke et al., 2010; Gerger and Leder, 2015). One of the studies showed this for representational paintings (Belke et al., 2010). But when comparing the group with matching titles and the group with unexplained non-matching titles to the control condition that did not receive any titles, it can be concluded that this effect was mainly driven by representational paintings being less liked due to the unexplained mismatch of title and content than paintings being more liked due to a match between the title and content. In other words, while the high liking typical for representational art is not easily enhanced by titles, unexplained inconsistencies such as mismatching titles can substantially decrease the liking of representational art. The authors (Belke et al., 2010; Gerger and Leder, 2015) assume that the reduced liking is caused either by lower processing fluency and meaning making or by a reduced understanding of the painting. Higher liking in contrast is assumed to be caused by better understanding, higher processing fluency and disfluency reduction.

That unexplained inconsistencies can lead to a disfluent processing is supported by research outside the field of aesthetics. For example, discrepancies between a picture of a map and a related text led to longer fixation times on the text and the picture of the map than text and picture of the map providing similar information. This was interpreted as a hampered process of information integration (Schüler, 2017). Disfluency due to unexplained inconsistencies might not only arise when additional information does not match the content of a picture or painting but also when the content of a seemingly realistic representational painting does not match reality. Historical paintings frequently contain historical inaccuracies, which are inconsistencies between the depiction of a historic event in a painting and a more plausible version of the event based on today’s historians’ opinions (Burke, 2001). Museums of art and history often provide additional information in the form of audio-texts naming the paintings inaccuracies. Without high background knowledge, the inaccuracies cannot be seen or inferred by looking at the painting. Therefore, mentioning them in the form of additional information is important for interpreting and understanding the painting. However, this could affect art processing and the evaluation of the representational artwork especially when the inconsistencies remain unexplained.

## Art Processing, Aesthetic Emotions, and the Evaluation of Artworks

The studies on the subjective understanding and liking of art described in the previous section are mostly discussed in the context of two frameworks. First, the fluency theory (Reber et al., 2004) proposes that the easier the viewer’s processing, meaning making, and understanding of an artwork is, the more the artwork will be liked by the viewer. Fluency can thereby result from early processing stages such as the classification of the artwork or the perceptual analysis of symmetry but also from later higher order processing stages, such as the cognitive mastery of an artwork. Similar to the hypothesis of the fluency theory is the simplified hypothesis derived from the psycho-historical framework for the science of art appreciation (Bullot and Reber, 2013). The psycho-historical framework states that higher understanding of an artwork is positively linked to its aesthetic liking. In both frameworks, additional information, such as titles or explanations of the style and the art historical context, is assumed to enhance but also lower the liking of artworks, depending on whether the additional information contributes to a fluent processing and better understanding or to a less fluent processing and lower understanding.

The two frameworks have been recently incorporated into a more complex theoretical model proposing positive and negative effects of additional information on the subjective understanding and liking of artworks as well as aesthetic emotions. These outcomes are assumed to mainly depend on two cognitive appraisals made during the higher order processing stage of cognitive mastery of an artwork. The Vienna integrated model of art perception (VIMAP) (Pelowski et al., 2017) proposes seven stages of art processing. The first stage is the pre-classification, which includes factors of context (museum, laboratory, social or individual setting) and personal factors (mood, personality, and expectations) that influence the viewers processing and emerge before a person deals with an artwork. In the second stage, the perceptual analysis, the low-level features of an artwork are processed, such as complexity, contrasts, and color. In the third stage, the implicit memory integration, elements of the painting are combined to more or less meaningful patterns. Thereby, factors such as familiarity and prototypicality play a role. In the fourth stage, the explicit classification, viewers identify the content in accordance with the painting’s context, style, and information learned about the artist. In all these stages, the focus is mainly on bottom-up processes that influence the art perception of a viewer.

For effects of additional information on subjective understanding and liking, especially the fifth stage, the cognitive mastery, is important. Cognitive mastery is characterized by top-down processes that consider and combine the information gathered by the bottom-up processing in order to form coherent meaning of the artwork together with an appropriate evaluation and physical response. The outcome of this mastery process depends on two processing checks: schema congruency check and self-relevancy check. For the schema congruency check, viewers consider their schemas about their knowledge, expectations, understanding, and opportunities for learning (Silvia, 2009). Thereby they also consider the success of the processing during the former stages of basic perceptual processing, object identification, explicit classification, and integrating these elements. The match for each of these elements can be more or less congruent. A good overall match results in a subjective feeling of fluency and an efficient processing and understanding. For example, viewers could check whether their understanding of the artwork matches the level of understanding they expected. The second check proposed by the model is the self-relevancy check. With this check, the viewers consider the personal importance of the artwork for their self-image. The viewers decide whether the outcome of their viewing is relevant to them and whether they really have an interest or need to process the artwork. Stage six, the secondary control, is only relevant if viewers experience low congruency and high self-relevancy and cannot resolve the incongruency by direct mastery. In this stage, viewers try to reduce the incongruency by different strategies. They can re-classify the artwork or the context by reducing the importance of the incongruent artwork. They can also just leave the gallery physically to escape the experience of incongruency. Stage seven, the metacognitive self-reflection only takes place if viewers cannot disengage from viewing the artwork during stage six. In this stage, the viewers think about the difficulties in processing the artwork and reflect on expectations and failed attempts to master or reduce the incongruency. This will allow for a new and likely more harmonious approach in processing the artwork.

The self-relevancy check and therefore stages 6 and 7 are only relevant for experts in real art situations. When considering laypersons outcomes of art processing, the self-relevancy check can be neglected, since art processing does rarely threaten their self-image. The model then suggests two different outcomes based on the congruency-check during the cognitive mastery stage. The first outcome results from high schema congruency together with low self-relevance. It is characterized by a default or facile reaction. This is probably the most common outcome of viewers not finding something new or questioning in the artwork. The result is a sufficient classification, easy processing and understanding of the artwork with little emotional engagement, and a facile feeling of pleasure. The second outcome results from low schema congruency together with low self-relevance. It is characterized by a reaction of novelty and small insight due to a small incongruency in the congruency-check. Certain aesthetic emotions are thereby triggered, depending on whether or not the viewers are able to resolve the incongruency. Viewers have different option to resolve the incongruency: (a) Viewers can resolve incongruency by continuing their processing to find more information that contributes to a higher match. (b) Viewers can render the incongruency as irrelevant. (c) Viewers can modify their schema by generalizing definitions, classes, or expectations to include the novel elements. (d) Viewers can accept the incongruency as a mystery and accept the ambiguity and not seek a resolution. (e) Sometimes the incongruency is explained by further additional information. In these cases, the viewers can rate the chance high that incongruencies are resolved and might find pleasure and interest for the incongruency. Alternatively, the viewers appraise the chance of finding a resolution to be low and experience a need for a resolution in order to restore coherence. This will result in confusion, in lower interest, and probably in a lower subjective understanding and liking of the artwork. Hence, confusion and interest are opposite outcomes of the same cause, depending on the viewer’s appraised chance to form a coherent understanding after an incongruency was encountered (cf. Silvia, 2009). Independent of the appraised chance to solve the incongruency, all viewers should experience surprise when confronted with incongruency.

Empirical evidence of effects of additional information on aesthetic emotions is scarce. The present literature has mostly reported no effects of additional information in the form of titles on the emotional experience (Bubić et al., 2017) and interest (Leder et al., 2006; Gerger and Leder, 2015). One study considered art appreciation as a scale of interest and liking ratings together and reported significant effects of additional information (Swami, 2013). To the best of our knowledge, no studies have investigated the effects of additional information on surprise and confusion. Therefore, we found there was a need to empirically test the assumptions of the VIMAP with regard to emotional outcomes. We thereby expected not only that an incongruency in the congruency-check can arise from inconsistencies, such as titles semantically mismatching the content, but also by naming the historical inconsistencies of the content of a representational painting mismatching reality.

## Transportation as a Result of Processing a Narrative Artwork

As we used historical paintings in our study that depict a story and are therefore inherently narrative, we also considered theories of narrative processing to investigate the influence of additional inconsistent information on processing outcomes. According to the model of narrative comprehension and engagement (Busselle and Bilandzic, 2008), the fluent processing of a narrative, presented either in the form of texts, films, or pictures, leads to the feeling of being transported into the story. The phenomenon of being transported is described as the readers’ or viewers’ experience of being mentally absorbed in the story world (Gerrig, 1993) and consists of a cognitive, emotional, and imaginary component (Green and Brock, 2002). Transported individuals focus their cognitive processing on the events of the story; they identify and feel with the characters and create vivid mental images of the places and characters. They can experience a flow-like state and lose awareness of what is going on around them (Green and Brock, 2000). Transportation is enjoyed by the recipients (Busselle and Bilandzic, 2008; Bilandzic and Busselle, 2011) and is therefore an essential experience also when processing narrative artworks. Transportation was mostly investigated with written text and movies but is assumed to apply to narratives presented in all modalities (Green and Brock, 2000). The model of narrative engagement assumes that readers and viewers fluently process and experience transportation when the story is coherent. However, when the recipients encounter incoherence or implausibilities that are not explained by the story world, processing fluency is diminished and transportation is lowered (Busselle and Bilandzic, 2008). This link between perceived realism and transportation is supported by empirical results (Green, 2004; Bilandzic and Busselle, 2011). Based on the model of narrative comprehension and engagement (Busselle and Bilandzic, 2008) and the related empirical studies, we expected that naming a paintings’ inconsistencies reduces transportation and that explaining these inconsistencies by benevolent intentions of the painter will compensate for this negative effect.

## The Present Study

In our study, we investigated the effects of additional information naming a painting’s inconsistencies on the viewers’ art evaluation and aesthetic emotions when viewing representational art, that is, in specific historical paintings. Based on the VIMAP and related empirical findings (Belke et al., 2010; Gerger and Leder, 2015), we expected that the naming of a painting’s inconsistencies and leaving them unexplained lowers the subjective understanding and the liking of the historical painting. Additionally, we assumed that informing the viewers about the artists’ intentions in order to explain these inconsistencies can help the viewer to restore coherence. Hence, the information about the artists’ intentions should compensate for the negative effects of naming inconsistencies without explaining them. This should be manifest in a two-way interaction between the factors naming of inconsistencies and explanation for subjective understanding (P1) and liking (P2) of the historical paintings. Based on the VIMAP, we further expected that surprise will generally be higher with inconsistencies named compared to without inconsistencies named, indicated by a main effect of naming inconsistencies (P3). Additionally, the viewers should experience lower interest and higher confusion with inconsistencies named compared to without inconsistencies named when no explanation is given, but these effects on interest (P4) and confusion (P5) should be compensated for by the provision of an explanation about the artists’ intentions. Lastly, based on the model of narrative comprehension and engagement (Busselle and Bilandzic, 2008), we expected that naming the painting’s inconsistencies without explaining them reduces the viewers experienced transportation compared to not naming and explaining the inconsistencies, but explaining these inconsistencies by mentioning the artists’ intentions should compensate for this effect (P6). Hence, for Predictions 4, 5, and 6 we again predicted two-way interactions between the factors naming of inconsistencies and explanation.

### Method

#### Participants

The experiment was done online and could be accessed with all common browsers. We recruited 196 participants on Prolific and instructed them only to participate via computer or tablet and not via smartphone due to the small screen size, which we considered insufficient for noticing the details of the paintings and for an appropriate aesthetic experience of the paintings. The available participants were pre-filtered to include only native speakers of German. From the 196 participants, 41 participants were excluded because they already knew at least one of the three paintings we used in the present study. Four were excluded because they participated via smartphone. Six were excluded because they gave 50% or less correct answers in a memory check, indicating that they had guessed the answers and had not listened closely to the audiotexts commenting on the picture. The memory check presented several statements about the historic event, for example, that it was summer when Washington crossed the Delaware and asked if this was depicted in the painting or not. The painting clearly depicts floating ice floes on the river, and the audio-texts also states that it was winter. Four participants were excluded because they studied or worked in the field of history or art-history. Subsequently, 139 participants remained for the analysis: 64 (46%) females, 75 (54%) males; aged between 18 and 67 years (*M* = 30.78, *SD* = 9.95).

#### Design

We tested our predictions using a 2 × 2 design with naming of inconsistencies (with vs. without) and explanation (yes vs. no) as the between-subjects factors. The 139 participants were randomly assigned to one of our four conditions (I−E−: without inconsistencies named and no explanation provided, *n* = 40; I+E−: with inconsistencies named and no explanation provided, *n* = 30; I−E+: without inconsistencies named and an explanation provided, *n* = 40; I+E+: with inconsistencies named and an explanation provided, *n* = 29).

#### Material

As research material, we used pictures of three historical paintings: “Valdemar Atterdag Holding Visby to Ransom” by Carl Gustaf Hellqvist, “The Death of General Wolfe” by Benjamin West, and “Washington Crossing the Delaware” by Emanuel Leutze. All of these paintings contain pictorial elements that are consistent and pictorial elements that are inconsistent with a plausible version of the historical event based on today’s historians’ opinions.

We created four different versions of audio-texts for each painting, depending on the respective condition. The audio-texts in all conditions commented on eight pictorial elements for each painting. This consisted of information about the location of the pictorial element in the painting, its description, and an interpretation of the element regarding the historic event. The audio-text’s interpretation of four pictorial elements of each painting was manipulated according to the condition. These elements were either named as being inconsistent to the actual historic event (with inconsistencies named) or not (without inconsistencies named). Directly after this, either information about the intention of the artist followed that was able to explain the inconsistency (explanation provided) or a text of similar length and verbal content followed that did not inform the participants about the intention of the artist and did not explain the inconsistency (no explanation provided). The information about the artists’ intentions was formulated in a way that made sense even when no inconsistencies were named. The intention of the artist was always benevolent, for example, by stating that the artist wanted to make a certain point clearer to the viewer (see Table 1 for an audio-text example). The audio texts had different durations for the paintings (4:29 min for Hellqvist, 4:41 min for Leutze, 4:35 min for West) but were of equal length for the four conditions with only minor changes in the sentences.

**TABLE 1:** Example of text for the native American in wests painting “the death of general wolfe” for the four conditions.

On the left kneels a Native American wearing loincloth and a red feather. It is one of the Iroquois who were allied to the British. The Iroquois were engaged as scouts before the battle	
***Without inconsistencies named (I−)***	***With inconsistencies named (I+)***

During the combat they did indeed leave the camp and took part in the battle.	During the combat they did not leave the camp and did not take part in the battle.
***Without explanation (E−)***	***With explanation (E+)***

Benjamin West painted the picture in London 11 years after the event for an English audience. The appearance of the North American Iroquois was not very well known at that time, and the viewers could therefore not recognize North America as the place of the action in West’s painting.	Benjamin West painted the picture in London 11 years after the event for an English audience. The appearance of the North American Iroquois was already very well known at that time and West helped the viewers to recognize North America as the place of action with his depiction.

*Intention of the artist explaining the discrepancy is underlined. Either the inconsistency was named or not, then either an explanation followed or not.*

### Measures

To control for *a priori* differences between the conditions, we measured the participants’ general interest in art using the respective part of the German version of the Vienna Art Interest and Art Knowledge Questionnaire (Specker et al., 2018). Participants answered the questions on a seven-point Likert scale ranging from one (not at all) to seven (completely) for their self-reported interest and from one (less than once a year) to seven (once a week or more) for their self-reported activities in the context of art. We calculated the mean score for general interest in art. The internal consistency of the general interest in art scale was good as indicated by a Cronbach’s Alpha of α = 0.88.

We measured the subjective understanding for each painting with a two-item scale. Answers had to be given on a seven-point Likert scale ranging from one (not at all) to seven (very much). These items were similarly used by Swami (2013) and adapted from Silvia (2005). We calculated the mean score of subjective understanding. The internal consistency of the subjective understanding scale was good, as indicated by a Cronbach’s Alpha of α = 0.88.

We measured liking, surprise, interest, and confusion for each of the three paintings with the two items of the respective sub-scales of the German Version of the Aesthemos scale (Schindler et al., 2017). We used the original instruction of the Aesthemos to focus the participants on their own aesthetic experience. The instruction states in German: “Welche gefühlsmäßige Wirkung hatte x auf Sie? Bitte kreuzen Sie zu jedem Gefühl unten die Kategorie an, die auf Ihr persönliches Erleben am besten zutrifft. Bitte geben Sie nur an, wie Sie sich tatsächlich gefühlt haben. Beschreiben Sie nicht die Gefühle, welche im zuletzt gesehenen Gemälde ausgedrückt wurden, wenn Sie diese nicht selbst empfunden haben. [Which emotional effect did x have on you? For each emotion listed below, please mark the response category that best matches your personal experience. Please only indicate how you actually felt. Do not characterize the emotions expressed in x if you did not feel them yourself].” We replaced x with “das zuvor gesehene Gemälde [the previously seen painting].” For each emotion (liking, interest, confusion, surprise), answers were given on two items on a five-point Likert scale ranging from one (not at all) to seven (very much). The two items of liking were “Empfand ich als schön [I found it beautiful]” and “Gefiel mir [I liked it].” We calculated the mean scores of liking, interest, confusion, and surprise. The internal consistency of all subscales of the Aesthemos were acceptable to good as indicated by Cronbach’s Alphas of α = 0.85 for liking, α = 0.88 for interest, α = 0.73 for confusion, and α = 0.80 for surprise.

We measured transportation into the historic event with the adapted version of the six-item transportation short-scale (Appel et al., 2015) after each painting. For item five and six stating “While viewing the painting, I could imagine […] vividly,” we inserted one of the manipulated pictorial elements into the gaps, for example, “Fraser wearing a kilt” for the West painting. We calculated the mean score of transportation. The internal consistency of the transportation scale was excellent as indicated by a Cronbach’s Alpha of α = 0.92.

### Procedure

At the beginning of the experiment, the participants were instructed to focus on the paintings as artworks. For this, they were informed that they will see three paintings, of which the originals are exhibited in museums. Therefore, they can imagine the study to be similar to a visit in an art museum. The participants were further informed that the paintings depict historic events and that an accompanying audio-text will present further information about the painting, the artist, and the historic event. They were instructed that the audio-text for each painting could only be listened to once, and after viewing the paintings, they will be asked questions about the paintings. After this, the participants were asked about their general interest in art. Before the presentation of the paintings, they were able to test and adjust their speakers with a short audio-text. Each painting was introduced by a written instruction of the title and the name of the artist. The participants clicked the continue-button when they had read the information. After this they had to click the play-button to start the presentation of the painting together with the respective audio-text. Directly after the presentation of each painting, the participants were asked to report their experienced transportation, their aesthetic evaluation including liking, surprise, confusion, and interest, and their subjective understanding of the painting. The presentation of the three paintings was done in random order to prevent order effects. After the presentation of all three paintings, the participants filled out their demographics, a question about prior knowledge of the paintings, and whether they work or study in the field of art, history, or art-history. They were then debriefed and paid 4.50 £. The study received institutional research ethics committee approval.

## Results

### Control Variable: General Interest in Art

A one-way analysis of variance (ANOVA) was calculated with condition (I−E− vs. I+E− vs. I−E+ vs. I+E+) as the between-subjects factor. The analysis revealed no differences in general art interest between the four conditions, *F*(3, 135) = 0.45, *p* = 0.721, η^2^*_p_* = 0.010. Therefore, differences between the conditions cannot be explained by differences in general art interest.

### Subjective Understanding

A two-way ANOVA was calculated across the three paintings with naming of inconsistencies (with vs. without) and an explanation of the intentions of the artist (yes vs. no) as between-subjects factors. The analysis revealed no significant main effect of naming inconsistencies, *F*(1, 135) = 0.79, *p* = 0.376, η^2^*_p_* = 0.006. Subjective understanding did not differ significantly with inconsistencies named (*M* = 5.27, *SD* = 0.99) compared to without inconsistencies named (*M* = 5.42, *SD* = 0.97). The main effect of explanation was not significant, *F*(1, 135) = 0.38, *p* = 0.540, η^2^*_p_* = 0.003. Subjective understanding did not differ significantly when an explanation was provided (*M* = 5.40, *SD* = 0.90) compared to when no explanation was provided (*M* = 5.32, *SD* = 1.05). In contrast to our expectations, the two-way interaction between naming inconsistencies and explanation was also not significant, *F*(1, 135) = 0.97, *p* = 0.326, η^2^*_p_* = 0.007. The Bonferroni-adjusted comparison showed neither a significant difference between with inconsistencies named (I+E−: *M* = 5.14, *SD* = 1.02) and without inconsistencies named (I−E−: *M* = 5.45, *SD* = 1.06) when no explanation was given, *p* = 0.186, nor between with inconsistencies named (I+E+: *M* = 5.41, *SD* = 0.96) and without inconsistencies named (I−E+: *M* = 5.39, *SD* = 0.88) when an explanation was given, *p* = 0.946.

### Liking

**FIGURE 1:**
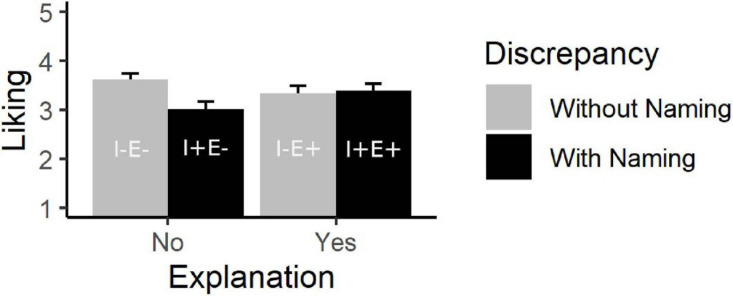
Interaction between discrepancy and explanation with regard to liking. The error bars indicate standard errors.

A two-way ANOVA was calculated across the three paintings with naming of inconsistencies (with vs. without) and an explanation of the intentions of the artist (yes vs. no) as between-subjects factors (see Figure 1). The analysis revealed no significant main effect of naming inconsistencies, *F*(1, 135) = 3.23, *p* = 0.074, η^2^*_p_* = 0.023. Liking did not differ significantly with inconsistencies named (*M* = 3.20, *SD* = 0.82) compared to without inconsistencies named (*M* = 3.47, *SD* = 0.91). The main effect of explanation was not significant, *F*(1, 135) = 0.11, *p* = 0.736, η^2^*_p_* = 0.001. Liking did not differ significantly when an explanation was provided (*M* = 3.36, *SD* = 0.89) compared to when no explanation was provided (*M* = 3.35, *SD* = 0.88). However, the two-way interaction between naming inconsistencies and explanation was significant, *F*(1, 135) = 4.89, *p* = 0.029, η^2^*_p_* = 0.035. As it was expected, the Bonferroni-adjusted comparison showed a significant lower liking with inconsistencies named (I+E−: *M* = 3.01, *SD* = 0.84) than without inconsistencies named (I−E−: *M* = 3.61, *SD* = 0.83) when no explanation was given, *p* = 0.005, but when an explanation was given, liking was equally high with (I+E−: *M* = 3.39, *SD* = 0.77) and without inconsistencies named (I−E+: *M* = 3.33, *SD* = 0.98), *p* = 0.771. To check whether the effect was similar across the paintings, we calculated an additional ANOVA including painting as a within-factor. The ANOVA with the three factors inconsistencies named, explanation, and painting revealed no significant three-way interaction *F*(2, 270) = 0.01, *p* = 0.994, η^2^*_p_* < 0.001. The two-way interaction was therefore valid for all three paintings. Further we checked the linkage between liking, subjective understanding and other aesthetic emotions. Liking ratings correlated positively with subjective understanding (*r* = 0.34, *p* < 0.001), interest (*r* = 0.71, *p* < 0.001), surprise (*r* = 0.54, *p* < 0.001), and also transportation (*r* = 0.65, *p* < 0.001).

### Surprise

A two-way ANOVA was calculated across the three paintings with naming of inconsistencies (with vs. without) and an explanation of the intentions of the artist (yes vs. no) as the between-subjects factors. The analysis revealed no significant main effect of naming inconsistencies, *F*(1, 135) = 0.18, *p* = 0.669, η^2^*_p_* = 0.001. In contrast to our expectations, surprise did not differ with inconsistencies named (*M* = 2.37, *SD* = 0.82) compared to without inconsistencies named (*M* = 2.43, *SD* = 0.87). The main effect of explanation was not significant, *F*(1, 135) = 0.43, *p* = 0.512, η^2^*_p_* = 0.003. Surprise did not differ significantly when an explanation was provided (*M* = 2.35, *SD* = 0.80) compared to when no explanation was provided (*M* = 2.47, *SD* = 0.89). The two-way interaction between naming inconsistencies and explanation was not significant, *F*(1, 135) = 1.37, *p* = 0.245, η^2^*_p_* = 0.010. The Bonferroni-adjusted comparison showed neither a significant difference between with inconsistencies named (I+E−: *M* = 2.33, *SD* = 0.89) and without inconsistencies named (I−E−: *M* = 2.57, *SD* = 0.89) when no explanation was given, *p* = 0.258, nor between with inconsistencies named (I+E+: *M* = 2.41, *SD* = 0.76) and without inconsistencies named (I−E+: *M* = 2.30, *SD* = 0.84) when an explanation was given, *p* = 0.603.

### Interest

A two-way ANOVA was calculated across the three paintings with naming of inconsistencies (with vs. without) and an explanation of the intentions of the artist (yes vs. no) as the between-subjects factors. The analysis revealed no significant main effect of naming inconsistencies, *F*(1, 135) = 0.46, *p* = 0.499, η^2^*_p_* = 0.003. Interest did not differ significantly with inconsistencies named (*M* = 3.57, *SD* = 0.90) compared to without inconsistencies named (*M* = 3.46, *SD* = 0.99). The main effect of explanation was not significant, *F*(1, 135) = 0.01, *p* = 0.913, η^2^*_p_* < 0.001. Interest did not differ significantly when an explanation was provided (*M* = 3.52, *SD* = 0.94) compared to when no explanation was provided (*M* = 3.52, *SD* = 0.94). In contrast to our expectations, the two-way interaction between naming inconsistencies and explanation was not significant, *F*(1, 135) = 0.57, *p* = 0.453, η^2^*_p_* = 0.004. The Bonferroni-adjusted comparison showed neither a significant difference between with inconsistencies named (I+E−: *M* = 3.39, *SD* = 1.06) and without inconsistencies named (I−E−: *M* = 3.62, *SD* = 0.85) when no explanation was given, *p* = 0.311, nor between with inconsistencies named (I+E+: *M* = 3.53, *SD* = 0.94) and without inconsistencies named (I−E+: *M* = 3.52, *SD* = 0.96) when an explanation was given, *p* = 0.958.

### Confusion

A two-way ANOVA was calculated across the three paintings with naming of inconsistencies (with vs. without) and an explanation of the intentions of the artist (yes vs. no) as the between-subjects factors. The analysis revealed no significant main effect of naming inconsistencies, *F*(1, 135) = 0.19, *p* = 0.661, η^2^*_p_* = 0.001. Confusion did not differ significantly with inconsistencies named (*M* = 1.64, *SD* = 0.59) compared to without inconsistencies named (*M* = 1.59, *SD* = 0.58). The main effect of explanation was not significant, *F*(1, 135) = 0.09, *p* = 0.760, η^2^*_p_* = 0.001. Confusion did not differ significantly when an explanation was provided (*M* = 1.60, *SD* = 0.54) compared to when no explanation was provided (*M* = 1.63, *SD* = 0.62). In contrast to our expectations, the two-way interaction between naming inconsistencies and explanation was not significant, *F*(1, 135) = 0.07, *p* = 0.792, η^2^*_p_* = 0.001. The Bonferroni-adjusted comparison showed neither a significant difference between with inconsistencies named (I+E−: *M* = 1.67, *SD* = 0.68) and without inconsistencies named (I−E−: *M* = 1.60, *SD* = 0.58) when no explanation was given, *p* = 0.975, nor between with inconsistencies named (I+E+: *M* = 1.61, *SD* = 0.50) and without inconsistencies named (I−E+: *M* = 1.59, *SD* = 0.58) when an explanation was given, *p* = 0.707.

### Transportation

A two-way ANOVA was calculated across the three paintings with naming of inconsistencies (with vs. without) and an explanation of the intentions of the artist (yes vs. no) as the between-subjects factors. The analysis revealed no significant main effect of naming inconsistencies, *F*(1, 135) = 0.38, *p* = 0.540, η^2^*_p_* = 0.003. Transportation did not differ significantly with inconsistencies named (*M* = 4.19, *SD* = 1.13) compared to without inconsistencies named (*M* = 4.31, *SD* = 1.17). The main effect of explanation was not significant, *F*(1, 135) = 0.01, *p* = 0.917, η^2^*_p_* < 0.001. Transportation did not differ significantly when an explanation was provided (*M* = 4.26, *SD* = 1.16) compared to when no explanation was provided (*M* = 4.25, *SD* = 1.15). In contrast to our expectations, the two-way interaction between naming inconsistencies and explanation was not significant, *F*(1, 135) = 0.11, *p* = 0.739, η^2^*_p_* = 0.001. The Bonferroni-adjusted comparison showed neither a significant difference between with inconsistencies named (I+E−: *M* = 4.14, *SD* = 1.15) and without inconsistencies named (I−E−: *M* = 4.33, *SD* = 1.15) when no explanation was given, *p* = 0.501, nor between with inconsistencies named (I+E+: *M* = 4.23, *SD* = 1.13) and without inconsistencies named (I−E+: *M* = 4.29, *SD* = 1.19) when an explanation was given, *p* = 0.844.

## Discussion

Presently, evidence of the effects of additional information that is intended to foster meaning making on subjective understanding and liking of artworks is mixed (Chmiel and Schubert, 2019). Positive effects on liking are shown for highly abstract paintings but seldom for representational art (Leder et al., 2006; Moore and West, 2012; Swami, 2013). In two previous studies, negative effects on liking were found for unexplained mismatching titles, indicating that the processing of inconsistencies can lower the liking of abstract but also representational artworks (Belke et al., 2010; Gerger and Leder, 2015). Following these findings, we presented historical paintings together with additional information either naming their historical inconsistencies or not. In addition, we either provided an explanation for the inconsistencies or not. Based on the present results and theories of art processing (VIMAP; Pelowski et al., 2017), we expected that the naming of a historical paintings’ inconsistencies and leaving them unexplained would produce a similar effect like the unexplained mismatching titles. In both cases subjective understanding and liking of the artwork should be lower, due to the viewer’s processing of inconsistent information. In addition, we expected that the provision of an explanation would compensate for the negative effects of the naming of unexplained inconsistencies on subjective understanding (P1) and liking (P2).

Although subjective understanding was not significantly affected by the additional information in our experiment, liking was significantly lower when the inconsistencies of a painting were named but remained unexplained compared to when the inconsistencies were not just named, but also explained. In contrast, no similar difference in liking for accurate elements were found when explanations were provided or not provided. Because liking under conditions of naming and explaining inconsistencies was similar to conditions of not naming inconsistencies, we conclude that the explanations did compensate for the detrimental effects of naming inconsistencies. Our result of a lower liking when inconsistencies are named but not explained is in line with the assumptions of the fluency theory, VIMAP, and previous empirical evidence (Belke et al., 2010; Gerger and Leder, 2015). The result indicates that the negative effects in cases of missing explanations not only apply to semantically mismatching titles but also to longer explanations of representational art that require the viewer to process inconsistencies. Furthermore, our results show that the negative effects of unexplained inconsistencies on liking can be compensated for by an explanation of these inconsistencies. Hence, our study provides indication against the assumption that only the liking of abstract art can profit from additional information (Leder et al., 2006; Swami, 2013). In addition, it supports the claim that the reason for rare evidence of additional information affecting liking of representational art might be a frequent ceiling effect resulting from the already high liking of representational art (Chmiel and Schubert, 2019).

Our results of a significant two-way interaction between inconsistency named and explanation for liking without a significant two-way interaction for subjective understanding seem to be more compatible with the VIMAP than with the psycho-historical framework for the science of art appreciation. While the latter emphasizes the role of understanding on the liking of artworks the former assumes that liking is a product of the congruency-check, which includes understanding, but also other factors, such as whether the painting matches the viewers expectations. Also, in accordance with the fluency theory (Reber et al., 2004), a less fluent processing remains to be a possible explanation, that needs to be examined more directly with additional processing measures in future studies. Fluency was also considered to be the underlying mechanism in the studies using unexplained mismatching titles (Belke et al., 2010; Gerger and Leder, 2015).

Regarding aesthetic emotions, we expected that surprise will be higher when inconsistencies are named than when they are not named, independent of whether these inconsistencies are explained or not (P3). We expected that interest will be lower when inconsistencies are named but unexplained than when they are not named and explained and that this effect will be compensated by an explanation for the inconsistencies (P4). On the contrary, we expected that confusion will be higher when inconsistencies are named and unexplained than when they are not named and explained, and that this effect is compensated again by an explanation for the inconsistencies (P5). In contrast to our expectations, we could not show any effects of naming the painting’s inconsistencies on any of these emotional outcomes. Neither surprise nor interest were lowered, nor did confusion increase by informing the viewers about inconsistencies when no explanation was provided. Subsequently, we could also not show a compensating effect of explanations for interest and confusion. Research on aesthetics often reported non-significant effects of additional information, such as titles, on emotional outcomes (Bubić et al., 2017) or more specifically interest (Leder et al., 2006; Gerger and Leder, 2015). These results, however, are surprising since a close link between liking and the experience of aesthetic emotions can be assumed to be based on the models (Pelowski et al., 2017). Indeed, in our study, interest, surprise, and also transportation correlated significantly and highly positively with liking.

However, if our manipulation affected liking but neither subjective understanding nor aesthetic emotions, by which means was liking affected? In line with Gerger and Leder (2015) who found similar effects on liking but not on interest, we speculate that viewers based their lower liking on a greater disfluency in the condition in which inconsistencies were named and no explanation was given than in the conditions in which inconsistencies were not named. In addition, if inconsistencies were named but an explanation was given, the explanation may have reinstated fluency for subsequent processing. The explanation thereby may have compensated for the negative effect of naming unexplained inconsistencies on ratings of liking. This result is in line both with the fluency theory and the VIMAP. According to the VIMAP, this pattern would be expected if viewers base their liking judgments mainly on bottom-up processing (stage 2 to stage 4) such as fluency but do not engage in higher order cognitive processes. Because particularly laypersons tend to rely on lower stages of art processing for their evaluation of artworks (Mullennix and Robinet, 2018), we speculate that our manipulation affected art processing on lower stages but not on higher stages where understanding and aesthetic emotions would have been affected. Since audio explanations are intended to be used primarily by non-experts it would be interesting for future research to investigate whether and how audio explanations can also substantially affect laypersons higher order processing and thereby aesthetic emotions and the subjective understanding.

Based on theories of narrative processing (Busselle and Bilandzic, 2008), we expected lower transportation when unexplained inconsistencies are named than when they are not named, but this effect should be compensated for by an explanation (P6). We could not show that the naming of unexplained inconsistencies lowers transportation. Hence, our experiment provides no support for the model of narrative engagement (Busselle and Bilandzic, 2008) for static pictorial narratives. Moreover, the effects on liking in our study are not comparable to effects on transportation being associated with enjoyment.

Some limitations must be noted: For aesthetics, we relied solely on self-reports. Physiological or other process measures, however, could be helpful as additional indicators of emotions, liking, and the possibly less fluent processing. For spoken text, it might be important to consider not only what additional information is presented, but also how it is presented. In our study, we stressed the words similarly whether inconsistencies were presented or not. In a realistic context, surprising facts can be presented with a voice emphasizing this surprise which could foster effects of additional information on aesthetic emotions. Further, regarding emotions, it might be that the between-design of our study with participants either always viewing inaccurate paintings or participants always viewing accurate paintings prevented effects of the naming of unexplained inconsistencies. A within-design might be more suitable for investigating these effects on emotions. For example, Russell (2003) did not detect effects on aesthetic evaluation in the first experiment by using a between design but did in the second experiment by using a within design. Regarding the effects of an explanation of inconsistencies on liking, we always explained the inconsistencies by mentioning the good intentions of the artists. It would be interesting whether malevolent intentions would have similar effects or not. This could help to disentangle whether the effects result from the explanation itself or the additional positive information about the artist.

The reported effect sizes in previous studies for liking varied greatly in magnitude, depending on the information provided. Studies using titles as additional information often reported small effect sizes (Belke et al., 2010; Gerger and Leder, 2015), whereas high effect sizes are reported for content-specific information (Swami, 2013). As the additional information that we manipulated was a content specific interpretation and due to scarce previous studies on the effects of additional information on emotions, such as surprise and confusion, we decided to assume a medium effect of *f* = 0.25 for our study. Based on a power analysis using G-Power, 128 participants were required for a power (1-β) of 0.80 to detect a medium effect of *f* = 0.25 and α = 0.05. Due to our strict exclusion criteria, we had to exclude more participants than expected, and the remaining 139 participants were not equally distributed across the conditions, resulting in a sufficient but slightly lower power than we had aimed for. Regarding generalizability, we only considered laypersons of art. We would not expect the same results on liking for viewers more proficient in art and art-history due to their higher order processing or because they might be able to explain the inconsistencies themselves without the need for an external explanation by the audio-text (Bullot and Reber, 2013).

In conclusion, we could show that the naming of unexplained inconsistencies impairs the liking of representational paintings. However, an explanation about the inconsistency was able to compensate for this negative effect of additional information on the liking of representational artworks. Our results corroborate theories of art processing, such as the VIMAP (Pelowski et al., 2017), and show that not only abstract art can profit from additional information but also representational art. Our results extend the present literature by showing that negative effects of additional information hold not only for unexplained mismatching titles (Belke et al., 2015; Gerger and Leder, 2015) but also for informing about the inconsistencies of the content of a painting with regard to reality and at the same time leaving these inconsistencies unexplained. In contrast to unexplained mismatching titles, information about a painting’s historical inconsistencies is frequently provided in museums of art as this is an important part of the interpretation of a painting’s content. Therefore, our results have practical implications for the design of information accompanying representational artworks in museums. First, additional information can not only enhance the liking of artworks but also lower the liking of artworks if it requires a layperson to process unexplained inconsistencies. Second, if inconsistencies of a representational painting are first named unexplained, the liking can be restored when an explanation for the inconsistencies is added in a second step.

## Data Availability Statement

The raw data supporting the conclusions of this article will be made available by the authors, without undue reservation. Datasets are available on request.

## Ethics Statement

The studies involving human participants were reviewed and approved by Ethics Committee of the Leibniz-Institut für Wissensmedien. The patients/participants provided their written informed consent to participate in this study.

## Author Contributions

All authors contributed to writing the manuscript. MK performed the research and collected and analyzed the data.

## Conflict of Interest

The authors declare that the research was conducted in the absence of any commercial or financial relationships that could be construed as a potential conflict of interest.
